# The impact of teacher attitude and teaching approaches on student demotivation: Disappointment as a mediator

**DOI:** 10.3389/fpsyg.2022.985859

**Published:** 2022-09-07

**Authors:** Yeyao Tang, Jifan Hu

**Affiliations:** ^1^School of Digital Economics, Trade and Management, Chengdu Textile College, Chengdu, China; ^2^W. P. Carey School of Business, Arizona State University, Tempe, AZ, United States

**Keywords:** student demotivation, student disappointment, English language, teacher discouraging attitude, discouraging teaching approaches, knowledge hidings

## Abstract

Student demotivation with English as a medium of instruction has attracted increased attention of scholars, particularly in those countries where it is taught as a second or foreign language. While there is a consensus that proficiency in English brings several benefits, it is found that students are demotivated to learn in English. As a result, many studies have tried to investigate the factors that reduce the motivation to learn in English. Drawing on disappointment theory, this study aims to investigate why and how the discouraging attitude of a teacher and discouraging teaching approaches create negative emotions (i.e., disappointment with English as a medium of instruction), which in turn demotivates Chinese students with English as a medium of instruction. This study has adopted a cross-sectional research design. The data were collected through a self-administered questionnaire survey from Chinese students (*n* = 428) studying in different universities in China. The hypothesized relationships were tested through PLS-based SEM by using Warp-PLS 7.0 software. The findings show that teachers' discouraging attitudes and discouraging teaching approaches are important determinants of student demotivation with English as a medium of instruction. The results also confirm that student disappointment is an important underlying mechanism in the relationship between exogenous and endogenous variables. This study contributes to the literature on student demotivation with English as a medium of instruction by superficially identifying teacher-related factors in China, which were previously overlooked. The implications of this study are that the university administration should monitor the attitude of the teachers toward English, as well as their teaching approaches, in order to curb any potential threat to student motivation. The administration should train the teachers on how to show a positive attitude and follow encouraging teaching approaches. In this way, students' disappointment with the English language and teaching methodology can also be overcome, which will ultimately increase their motivation with English as a medium of instruction in universities.

## Introduction

English as a medium of instruction in educational institutions around the world has become an emerging trend, particularly in non-English-speaking countries. People learn English due to different reasons. In reality, English has become a global language and the most commonly used language for communication at the international level. Therefore, people, particularly parents, are putting extra emphasis that their children must learn and study in English. In this regard, proficiency in English has become an essential factor for the competitiveness of the students in the current globalized and modern society (Wu et al., 2020). Therefore, many governments from non-English-speaking countries are reforming their educational policies to adopt English as a medium of instruction (Kirkgoz, 2007), and are motivating students to study in English in order to meet the global challenges effectively (Koylu and Tracy-Ventura, 2022). For example, if a person wants to study in another country, or wants to do business at the international level, or wants to travel around the world, the only language that can help him or her to communicate with others is English. This suggests that adopting English as a medium of instruction in non-English-speaking countries is an effective strategy to reap the benefits of internationalization.

Despite significant efforts by the governments of non-English-speaking countries, it has been observed that the majority of the students, those who study English as a second language, are demotivated with English as a medium of instruction (Kim et al., 2014). Therefore, demotivation is “the gradual loss of motivation over a relatively long period (e.g., for weeks, months, or semesters, as opposed to within a single lesson) (Albalawi and Al-Hoorie, 2021, p. 1).” Demotivation with English as a medium of instruction has become an important area of inquiry that has attracted the increasing attention of scholars in recent years (Kim et al., 2014; Zhang et al., 2020). In this regard, most of the previous studies have focused on the reasons for demotivation, and have identified various external and internal factors that lead to this important problem (e.g., Kormos and Csizér, 2008; Kormos and Kiddle, 2013; Getie, 2020). Although existing research sufficiently enhances our understanding regarding the role of these factors in predicting students' demotivation, we argue that this stream of research is yet underdeveloped, particularly with reference to China, due to two important reasons.

First, English has gained an important role in the Chinese educational system (Hu, 2005). Chinese students start learning English from the primary level to the university level (Guo et al., 2020). The educational authorities in China are trying to make Chinese students proficient in English for several reasons, to effectively communicate with the global community, and to secure a good job position (Xiong and Yuan, 2018). However, Chinese students are found to be demotivated to learn English or to study English (Kim et al., 2014). For example, one factor that causes demotivation with English learning is Chinese students' belief in its usefulness, thereby they are less motivated to engage themselves in those classes that have English as a medium of instruction (Li, 2022). Zhang et al. (2020) found the experience of failure as an important determinant of demotivation with the English language among Chinese students. The teacher is also an important factor who can significantly impact the de(motivation) of students with English as a medium of instruction (Kikuchi and Sakai, 2009). In particular, teachers' discouraging attitudes and discouraging teaching approaches can cause demotivation to learn in English (Wang and Littlewood, 2021). However, existing research has not empirically tested the impact of these two teacher-related factors on students' demotivation with English as a medium of instruction in Chinese secondary schools, which is an important gap in the literature.

Second, although previous research has extensively focused on the impact of various factors (Kikuchi and Sakai, 2009; Vefali and Ayan, 2015) on the students' demotivation, very few studies have examined the underlying mechanisms through which these factors influence demotivation. Recently, researchers (i.e., Li, 2021) have highlighted the need for exploration of intermediating mechanisms between the relationship between different factors and students' demotivation to learn in English. This is also an important gap in the literature because the underlying mechanism helps us to understand how the effect of determinant factors transmits through mediating variables. This research gap creates the opportunity for researchers to conduct a comprehensive study into this important research area. Hence, this study intends to fill these research gaps in two important ways.

First, this study identifies teachers' discouraging attitudes and discouraging teaching approaches as important factors for students' demotivation (Takase et al., 2019). Existing research indicates that the attitude of a teacher is of great importance for students' motivation to learn (Ross-Hill, 2009). The attitude of a positive and encouraging teacher impacts positively on the student's attitude and behavior (Lee, 2019). In contrast, when a teacher has a discouraging attitude, it will more likely demotivate the students to learn English (Li, 2022). Similarly, teaching approaches also significantly impact the attitude and behavior of a person (Beausaert et al., 2013). Therefore, when a teacher adopts encouraging teaching approaches that create a sense of usefulness learning in the English language, the students will be more motivated to study English (Tran, 2013). Conversely, when the teaching approaches of teachers are discouraging, the students are more likely to be demotivated with English as a medium of instruction because they have negative feelings regarding the usefulness of studying in English (Chang and Hwang, 2018). In this way, we argue that both discouraging attitudes and discouraging teaching approaches are important determinants of students' demotivation with English as the medium of instruction in Chinese secondary schools. Hence, testing this impact empirically is an important contribution to the existing literature.

Second, drawing on the disappointment theory (Bell, 1985), this study considers students' disappointment, referred to as an “unpleasant feeling that arises when certain expectations are not realized” (Levering, 2000, p. 65), as an underlying mechanism. Teachers and students always expect from each other (Wubbels and Brekelmans, 2005). Therefore, when these expectations are not fulfilled, either by a teacher or by a student, disappointment may appear (Zhukov, 2013). Taking this into account, we argue that when students experience discouraging attitudes and discouraging teaching approaches by the teacher, they experience feelings of disappointment, which in turn leads to demotivation with English as a medium of instruction. This suggests that disappointment is an important mediating mechanism that can increase our insights into how different factors transmit their impact on students' demotivation. Therefore, this is another important contribution of the present research to the existing literature. The rest of the article is organized as follows. The next section covers the literature review followed by methodology, analysis, and results. The last section includes a discussion, theoretical and practical implications, limitations, future research directions, and a conclusion.

## Literature review

### The relationship between teacher discouraging attitude and student demotivation

Demotivation refers to a student's unwillingness to learn a second language, such as English (Dornyei and Ushioda, 2013). Demotivation results from external factors (Wang and Littlewood, 2021). Before this, existing of motivation is a prerequisite because its gradual decrease is later named demotivation (Cents-Boonstra et al., 2022). In other words, the absence of motivation can be named demotivation or motivation. A demotivated student feels that studying in English is useless because he or she is not unable to understand the lectures completely where the medium of instruction is English. Thereby, they feel demotivated (Falout et al., 2009). Though English is not new in China, however, in recent years English has received high importance in China due to the internationalization of the Chinese economy (Sundqvist and Olin-Scheller, 2013). English has become the symbol of success and status in China (Wu et al., 2020). People believe that proficiency in Chinese can open doors of opportunities for them (Wang and Littlewood, 2021). Therefore, parents in China are increasingly trying for those schools where English is a medium of instruction, or at least they have more focus on English (Dornyei and Ushioda, 2013).

Despite the importance of English as a medium of instruction, it has been noted that various factors can demotivate students to learn English. For example, an unfavorable learning environment, English as a compulsory subjective for non-natives, lack of confidence, and teachers' negative attitudes toward students' learning in English are some important factors that cause demotivation among students (Dornyei and Ushioda, 2013; Li, 2021). In addition to this, many other studies conducted in different Asian contexts (i.e., Japan and Korea) have identified various student, teacher, and context-related factors of demotivation (Kim et al., 2014; Zhang et al., 2020). Past research indicates that among other factors, the teacher is the most influential factor that can significantly impact motivation (Yadav and BaniAta, 2013). In particular, when a teacher has a discouraging attitude toward students or any language, such as English, students are less likely to show their interest to learn in that language. Consequently, students' motivation level goes down, and they feel demotivated. Taking this into account, except that when teachers negatively comment and show no interest in students or lack enthusiasm, the student will be more likely to be denotative with English as a medium of instruction (Takase et al., 2019). Hence, we propose the following hypothesis:

**Hypothesis 1:** *There is a positive relationship between teachers' discouraging attitudes and student demotivation with English as a medium of instruction*.

### The relationship between discouraging teaching approaches and student demotivation

As discussed earlier, many factors can demotivate the students to learn English. Teaching approaches or styles are also a key determinant of student demotivation (Hettiarachchi et al., 2021). It is found that students learn through various approaches, such as seeing, memorizing, observing, hearing, speaking, and doing (Visser et al., 2006). Therefore, teaching approaches also do vary (Aelterman et al., 2019). For example, some teachers deliver lectures by themselves or discuss a topic with the students, others provide reading materials, and some focus more on memorizing and practicing (Sampermans et al., 2021). The portion of learning a foreign language in the class can depend on students' native ability; however, in this regard, the most influential factor that can be a strong motivator and demotivate is the teaching approach of a teacher (Invernizzi et al., 2019).

A teaching approach refers to an individual's preferred style to teach (Visser et al., 2006). In literature, researchers categorized teaching approaches into student-focused approaches and content-focused approaches (Kember et al., 2002; Trigwell, 2012). When a teacher follows a teaching approach to teach the student in a specific context, it is referred to as a teacher-focused approach, and the objective is just to transmit the knowledge to the students (Trigwell, 2012). In addition, the teacher-focused approach particularly emphasizes students' organization, course content presentation, and lecture delivery in an understandable way. In contrast, student-focused approaches encompass students' facilitation, development of students' concepts, and knowledge development (Trigwell, 2012). In this approach, teachers try to develop students' preexisting concepts, motivate them to learn new ones, and encourage them to apply newly learned knowledge (Prosser and Trigwell, 1999).

Teachers' approaches to teaching the student play a major role in their engagement, motivation, and academic performance (Codina et al., 2018). Teaching approaches have been extensively discussed in the literature (Aelterman et al., 2019), particularly regarding learning a foreign language (Chetty et al., 2019). It is found in the literature that well-organized, supportive, and autonomy-oriented teaching approaches lead to various positive student-level educational outcomes, such as well-being, engagement, and motivation (Walsh et al., 2020). In contrast, highly controlling teaching approaches are found to lead to several negative outcomes, such as boredom, lack of interest, and demotivation (Trigwell, 2012). For example, some studies have found that when a teaching approach is considered discouraging due to irrelevant material, difficult to follow, and uninteresting, the students become less attentive, participative, demotivated, and poor performers (Sugano and Mamolo, 2021). This suggests that the teaching approaches of the teacher are significantly linked to the students' teaching outcomes. According to disappointment theory (Bell, 1985), people have expectations from each other, and their emotional, attitudinal, and behavioral responses are based on the fulfillment of those expectations. Teachers and students also have expectations from each other. Particularly, students expect their teacher to be component and teach in a way that would be interesting, logical, reason-based, easy to follow, and less demanding (Takase et al., 2019). Teaching approaches with these qualities are considered as encouraging teaching approaches that meet the expectations of students, thereby motivating them to learn a foreign language. The teaching approaches that lack these qualities are considered discouraging teaching approaches, which may lead to demotivation among the students. Taking this into account, we argue that teaching in a foreign language, such as English, is very demanding in the Chinese context; therefore, when a teacher adopts discouraging teaching approaches, students will be demotivated. Hence, we propose the following hypothesis:

**Hypothesis 2:** There is a positive link between discouraging teaching approaches and *student demotivation with English as a medium of instruction*.

### Disappointment with English language as a mediator

Disappointment is an unpleasant feeling that appears when someone fails to meet one's expectations (Levering, 2000). It is a common phenomenon, and almost everybody experiences it. In academic life, a student also has expectations from their teachers (Albers, 2009). In particular, the students are highly inspired by the teachers' personalities, attitudes, teaching styles, and behavior (Fernandez-Rio et al., 2017). The majority of students tend to follow their teachers when they inspire them (Alsharif and Qi, 2014). In contrast, when they find their teacher is not showing an appropriate attitude and is not adopting encouraging teaching approaches, they experience a feeling of disappointment (Bartholomew et al., 2018).

Previous literature indicates that successful learning in a second language, such as English, highly depends on teachers' attitudes (Mahfoodh, 2017). The attitude that a teacher shows during class to the students who are studying English (as a medium of instruction) can significantly influence students' emotions (Krischler and Pit-ten Cate, 2019). In other words, the type of attitude (negative or positive) of a teacher impacts the feelings, as well as their motivation accordingly (Ramirez, 2003). It is teachers' attitudes that can create pleasurable feelings, which in turn motivate them to learn more when English is used as the medium of instruction. In this regard, it is found that teachers' positive remarks about the benefits of studying current courses and their importance for future career prospects will facilitate the students to have more pleasurable feelings, and they become highly motivated to study that particular subject. This is quite reasonable because students adopt a particular field of study from a career perspective, and if their teachers have a negative attitude about the link between that subject of study and career, the students will be highly disappointed, thereby will be demotivated to learn in that subject (Malhotra et al., 2021). Moreover, it is often noted that teachers often lack enthusiasm for teaching (Ramirez, 2003), an important source of disappointment and demotivation.

As discussed earlier, students are inspired by their teachers, and when the students find their teachers less energizing, less enthusiastic, and have no interest in teaching, they will experience unpleasant feelings (i.e., disappointment with the English language), which in turn will demotivate them (Malhotra et al., 2021). Drawing on disappointment theory (Bell, 1985), this study takes student disappointment with the English language as an underlying mechanism between teacher attitude and student demotivation. We postulate that when a teacher has a negative attitude toward English as a medium of instruction, this will cause feelings of disappointment among students, which in turn demotivate the students from learning English. Therefore, we expect that disappointment with the English language can be an important mediating variable in the relationship between the discouraging attitude of teachers and student demotivation.

**Figure 1:**
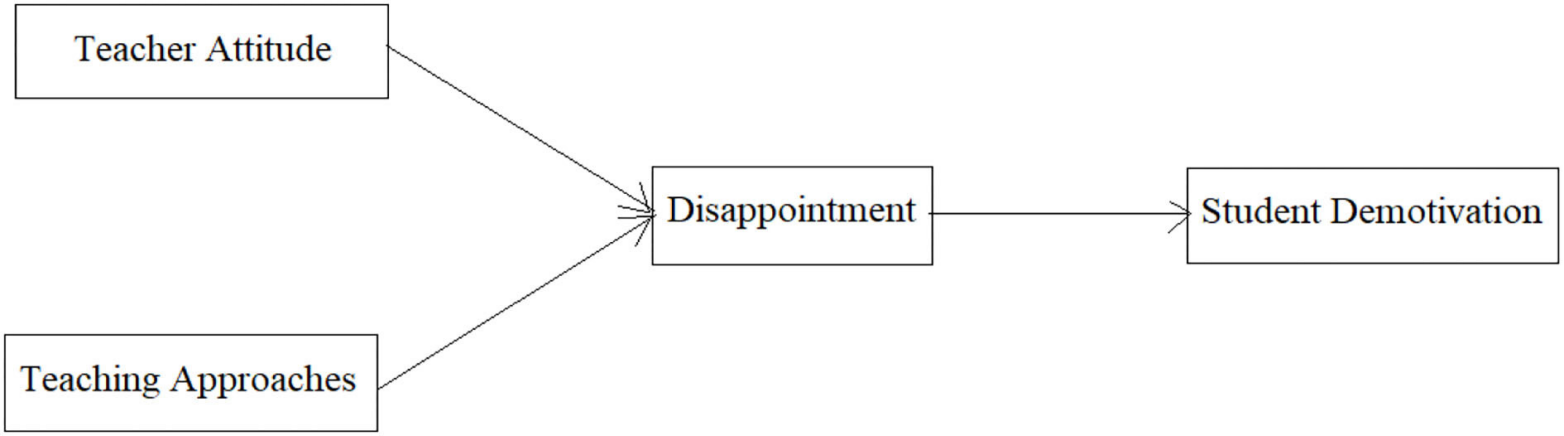
The theoretical framework.

Similarly, feelings of disappointment with the English language can also result from the teaching approaches of a teacher. Considering the importance of teaching approaches, it becomes very critical if students start feeling disappointed due to pedagogical issues (Levering, 2000). Characteristically, disappointment is an emotional experience that can lead to various negative outcomes (Rainey et al., 2009). When students' expectations are unfulfilled, they may experience several other negative feelings, such as demotivation (Towl, 2013). Students expect their teachers to come into the class with full knowledge of the subject, deliver the contents easily, and, understandably, involve the students in different learning activities, and give them feedback and advice (Ivanova and Korostelev, 2019). As a result, students feel happy with the teachers and are more motivated to learn. In contrast, when students find their teachers' approaches discouraging, they experience disappointment, which in turn demotivates them to learn that particular subject (Han and Yin, 2016). Based on the disappointment theory (Bell, 1985), we argue that students are very sensitive to the teaching approaches, and they expect that their teacher should follow those teaching approaches that will help them to gain mastery in that particular subject, because students select a subject due to its importance and usefulness in their career. When they find their teachers' approaches discouraging, they do not feel good and experience disappointment. As a result, they get demotivated. Taking this into account, we hypothesize that student disappointment with the English language can result from discouraging teaching approaches, which in turn diminishes their motivation level. Therefore, we expect disappointment with the English language as a mediator in the relationship between discouraging teaching approaches and student demotivation. Based on the hypothesis development and literature review following model (see Figure 1) has been formulated.

Hence, we formulate the following hypotheses:

**H3a:** *Disappointment with the English language mediates the relationship between teacher attitude and student demotivation*.

**H3b:** *Disappointment with the English language mediates the relationship between teaching approaches and student demotivation*.

## Methods

### Participants

A total of 550 students from 10 universities located in Beijing and Shanghai, China, participated in this survey study. In these universities, English as a medium of instruction is followed, and the Chinese students understand English. The majority of these students have already finished their compulsory course in English as a second language. Therefore, we find the respondents from these universities appropriate. We used the convenience sampling method. In the first step, the participants were briefed about the objective of the study and ensured that the data will be used purely for academic purposes. All the students participated in this survey voluntarily. Out of a total of 548 distributed questionnaires, we received back 428 with a response rate of 78%. In this sample, 257 (60%) were male students, and 171 (40%) were female students.

### Measures

#### Student demotivation

We adopted/adapted a five-item measure of student demotivation developed by Albalawi and Al-Hoorie (2021). The sample item includes “When I have a bad teacher, I lose interest and reduce the time I spend studying in English.” All the items were measured on a 5-point Likert scale ranging from strongly disagree (1) to strongly disagree (5). The Cronbach's alpha, of this measure, is 0.920.

#### Student disappointment with English language

Student disappointment with the English language was measured using a two-item measure by Albalawi and Al-Hoorie (2021). The sample item from this measure is “I am disappointed that spending a long time studying English at school was useless for speaking outside the university.” All the items were measured on a 5-point Likert scale ranging from strongly disagree (1) to strongly disagree (5). The Cronbach's alpha, of this measure, is 0.877.

#### Teacher discouraging attitude

To measure teachers' discouraging attitudes, we adopted/adapted statements from Takase et al. (2019). This measure consists of three items: “My teacher provides negative comments/feedback about my English,” “My teacher shows no interest in teaching in English,” and “My teacher shows no enthusiasm in teaching in English.” All the items were measured on a 5-point Likert scale ranging from strongly disagree (1) to strongly disagree (5). The Cronbach's alpha, of this measure, is 0.805.

#### Discouraging teaching approaches

To measure discouraging teaching approaches, we adopted/adapted a statement from Takase et al. (2019). In this study, Takase et al. (2019) used eight items to measure the construct of discouraging teaching approaches. The sample item includes “lack of chances to acquire new knowledge in a class where English is a medium of instruction,” “Lectures in English are difficult to follow,” and “Tasks in English are too demanding.” All the items were measured on a 5-point Likert scale ranging from strongly disagree (1) to strongly disagree (5). The Cronbach's alpha, of this measure, is 0.944.

## Analysis and results

To test the hypothesized relationships, this study used the variance-based partial least squares (PLS) SEM approach. For data analysis, Warp-PLS version 7.0 software was utilized. PLS is an appropriate method for data analysis because it works for small sample sizes, relaxes the normal distribution of data, and does theory testing (Kumar and Purani, 2018). Hence, this technique for data analysis is appropriate for this study.

### Common method bias test

To test the bias of the common method, we examined the full collinearity VIFs (Kock and Lynn, 2012). This test confirms the collinearity among the study variables (Kock and Lynn, 2012). It is recommended that the value of full collinearity (VIF) should be <5 (Kock, 2014). Hence, VIF <5 indicates that there is no issue of common method bias in the data (Hair et al., 2011). The analysis of this study shows that the values of full collinearity VIFs are less than the recommended value, which confirms that collinearity is not an issue in this study.

### Descriptive statistics

The descriptive statistics of the study variables are provided in Table 1. This table shows the minimum, maximum, mean, and values of standard divination.

**Table 1:** Descriptive statistics.

	***N***	**Minimum**	**Maximum**	**Mean**	**Std. deviation**
SDM	428	1.00	5.00	3.2084	1.03527
SDAP	428	1.00	5.00	2.7967	1.21256
DTAT	428	1.00	5.00	2.9611	0.92105
DTAP	428	1.00	5.00	3.2097	1.04715
Valid N	428				
(listwise)

SDM, Student demotivation; SDAP, Student disappointment; DTAT, Discouraging teacher attitude; DTAP, Discouraging teaching approaches.

### Measurement model

All the constructs of this study in the measurement model are reflective variables. The assessment of the outer model was based on three important criteria: composite reliability, discriminant validity, and convergent validity. The findings show appropriate levels of discriminant and convergent validity. For convergent validity, the rule of thumb is that the outer loading must be >0.70 (Chin, 1995). Table 2 shows that the outer loading for each item meets this criterion, hence confirming the existence of convergent validity. For discriminant validity, we analyzed the cross-loadings of each construct. The findings show that the indicator of each measure was highly correlated, whereas they were not correlated with the items of other measures, and we did not find overlapping of the indicators (see Table 2). Further, in Table 3, the square root of AVE for all measures is higher than the threshold criteria of 0.50 (Fornell and Larcker, 1981). This confirms the discriminant validity.

**Table 2:** Combined and cross-loadings for the outer model.

**Indicators**	**SDM**	**SD**	**DAT**	**DTAP**	**Type (as defined)**	**SE**	***p*-value**
SDM1	**(0.865)**	0.011	0.036	−0.002	Reflective	0.043	<0.001
SDM2	**(0.874)**	−0.051	0.033	0.069	Reflective	0.043	<0.001
SDM3	**(0.864)**	−0.034	−0.070	−0.070	Reflective	0.043	<0.001
SDM4	**(0.871)**	0.024	0.039	−0.050	Reflective	0.043	<0.001
SDM5	**(0.879)**	0.048	−0.038	0.052	Reflective	0.043	<0.001
SD1	−0.008	**(0.944)**	−0.056	0.035	Reflective	0.043	<0.001
SD2	0.008	**(0.944)**	0.056	−0.035	Reflective	0.043	<0.001
DTA1	0.014	0.011	**(0.874)**	0.031	Reflective	0.043	<0.001
DTA2	−0.012	−0.031	**(0.841)**	0.067	Reflective	0.043	<0.001
DTA3	−0.003	0.019	**(0.829)**	−0.101	Reflective	0.043	<0.001
DTAP1	0.054	−0.062	−0.052	**(0.842)**	Reflective	0.043	<0.001
DTAP2	0.037	0.037	−0.043	**(0.868)**	Reflective	0.043	<0.001
DTAP3	0.000	−0.040	0.038	**(0.833)**	Reflective	0.043	<0.001
DTAP4	−0.038	0.076	−0.035	**(0.846)**	Reflective	0.043	<0.001
DTAP5	−0.009	0.039	−0.015	**(0.812)**	Reflective	0.043	<0.001
DTAP6	−0.030	−0.019	0.044	**(0.858)**	Reflective	0.043	<0.001
DTAP7	−0.048	−0.009	0.051	**(0.854)**	Reflective	0.043	<0.001
DTAP8	0.033	−0.021	0.011	**(0.873)**	Reflective	0.043	<0.001

STDM, Student demotivation; SDAP, Student disappointment; DTAT, Discouraging teacher attitude; DTAP, Discouraging teaching approaches.

Values in bold shows the relationship and significance in variables.

**Table 3:** Correlations and the square root of AVEs.

	**SDM**	**SDAP**	**DTAT**	**DTAP**	**Composite reliability**	**Cronbach's alpha**	**Full collinearity VIFs**
SDM	**0.871**				0.940	0.920	1.212
SDAP	0.304	**0.944**			0.942	0.877	1.213
DTAT	0.322	0.307	**0.848**		0.885	0.805	1.230
DTAP	0.296	0.313	0.320	**0.848**	0.954	0.944	1.216

Square roots of average variances extracted (AVEs) are shown on diagonal.

STDM, Student demotivation; SDAP, Student disappointment; DTAT, Discouraging teacher attitude; DTAP, Discouraging teaching approaches.

Values in bold shows the relationship and significance in variables.

Table 3 shows the values of composite reliabilities and the values of internal consistencies of all measures. It is recommended that values for both should be >0.70. The findings indicate that all the values meet this criterion, hence confirming the reliability (Hair Jr et al., 2016).

### Structural model

**Figure 2:**
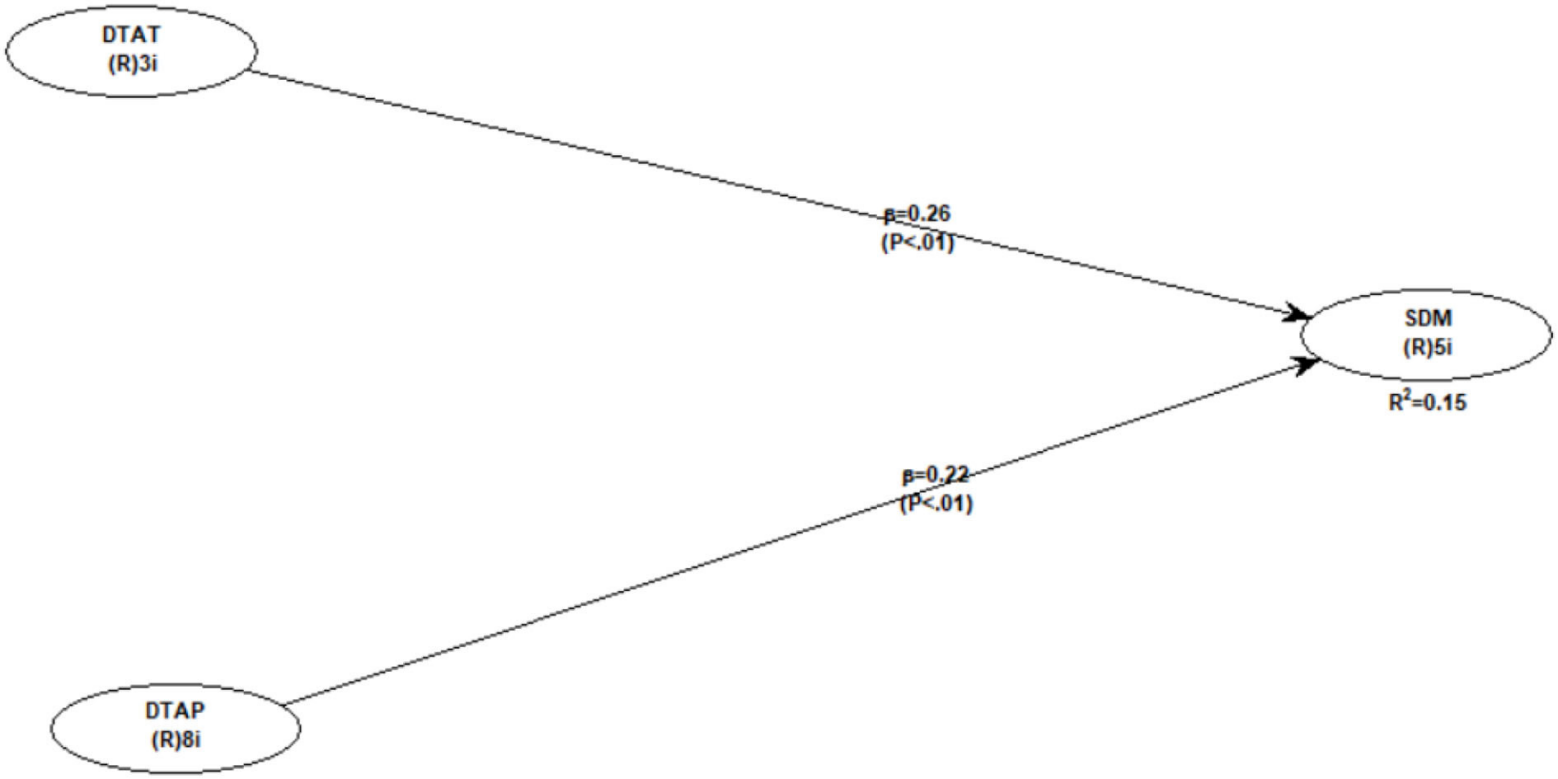
Direct model.

To test the hypothesized relationship between variables, we performed PLS-SEM to calculate the path coefficients and their respective *p*-values. Figure 2 presents the path analysis of this study. The findings of the path analysis are assessed based on different model fit and quality indices (Kock, 2014). Model fitness indices show a good fit to the data (see Table 4), which allows us to proceed with the hypothesis testing.

**Table 4:** Model fit and quality indices.

**Parameters**	**Value**	**Criteria**
Average path coefficient (APC)	0.214, *P* < 0.001	-
Average R-squared (ARS)	0.170, *P* < 0.001	-
Average adjusted R-squared (AARS)	0.165, *P* < 0.001	-
Average block VIF (AVIF)	1.153	acceptable if ≤ 5, ideally ≤ 3.3
Average full collinearity VIF (AFVIF)	1.217	acceptable if ≤ 5, ideally ≤ 3.3
Tenenhaus GoF (GoF)	0.362	small ≥ 0.1, medium ≥ 0.25, large ≥ 0.36
Sympson's paradox ratio (SPR)	1.000	acceptable if ≥ 0.7, ideally = 1
R-squared contribution ratio (RSCR)	1.000	acceptable if ≥ 0.9, ideally = 1
Statistical suppression ratio (SSR)	1.000	acceptable if ≥ 0.9, ideally = 1
Nonlinear bivariate causality direction ratio (NLBCDR)	1.000	acceptable if ≥ 0.9, ideally = 1

While testing the hypotheses, we first performed a direct path analysis, which represents hypotheses 1 and 2. As shown in Table 5, a teacher's discouraging attitude has a positive and significant impact on student demotivation (β = 0.256; *p* <0.001). Similarly, discouraging teaching approaches also significantly and positively impact student demotivation (β = 0.220; *p* <0.001). Hence, both direct hypotheses of this study are supported. Figure 2 represents the output of the direct path model.

**Table 5:** Direct path coefficient and *p*-values.

**Direct Path**	**β**	***p*-value**	**Remarks**
Teacher discouraging attitude	0.256	<0.001	Supported
→ Student Demotivation
Discouraging teaching approaches	0.220	<0.001	Supported
→ Student Demotivation

**Figure 3:**
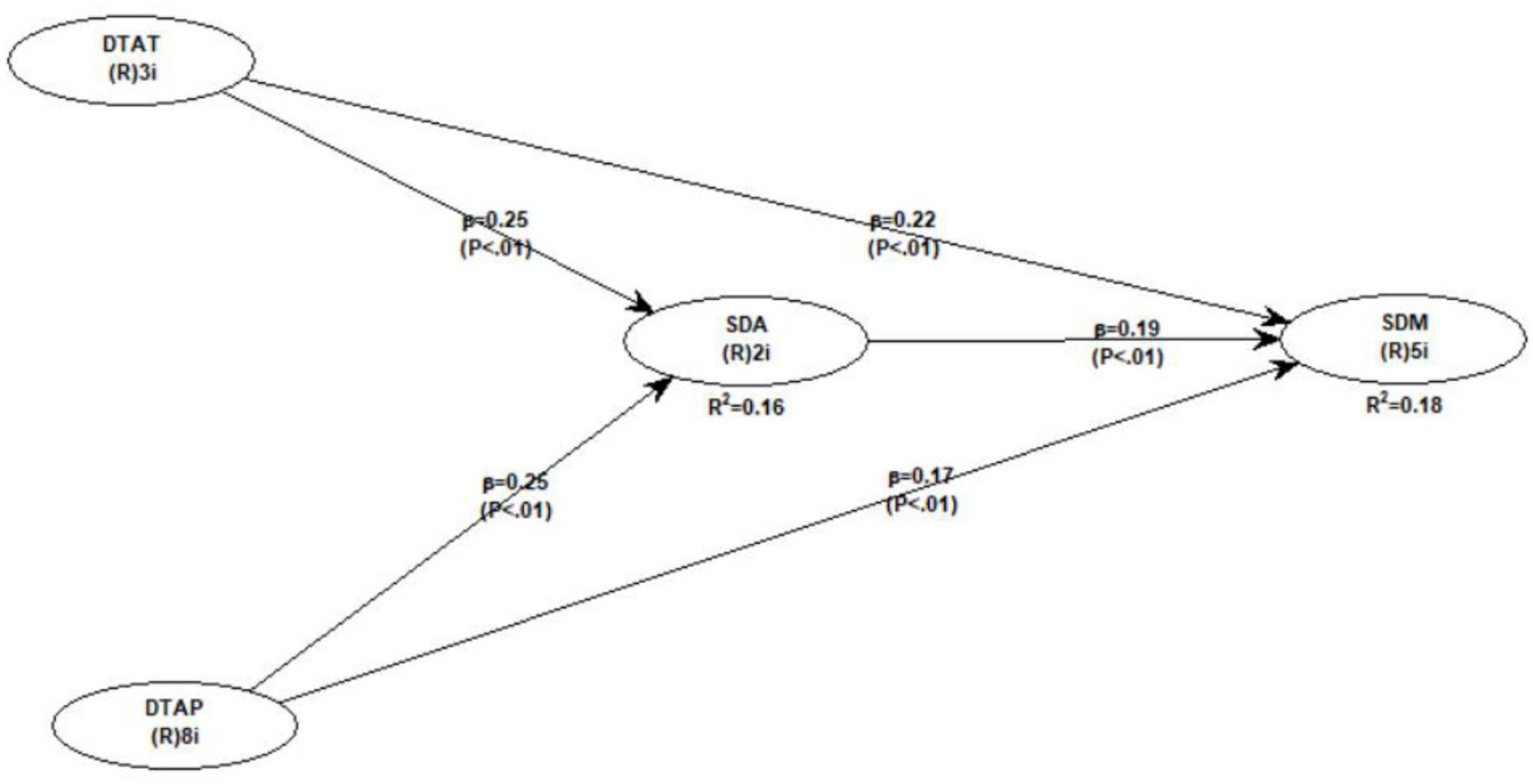
Indirect model.

Since we also proposed hypotheses regarding the indirect (mediating) impact of teacher discouraging attitudes and discouraging teaching approaches on student demotivation, we also performed an indirect model by containing the direct path, as well to assess the mediation effects (see Figure 3). Tables 6, 7 shows the results of indirect effects. As shown in the table, when we enter the mediating variable of student disappointment in the indirect model, the direct impact of both independent variables (i.e., teacher discouraging attitude and discouraging teaching approaches) is reduced but remains significant. Additionally, we further analyzed the direct, indirect, and total effects with their respective effect sizes, which were medium for the confirmation of mediation effects. This indicates the existence of partial mediation. Hence, it is confirmed that student disappointment is an important underlying mechanism. Therefore, hypotheses 3a and 3b are supported.

**Table 6:** The mediating role of student disappointment.

**Variable**	**Path to student disappointment**	**Path to student demotivation**
Teacher discouraging attitude	0.248 (*p* < 0.001)	0.216 (*p* < 0.001)
Student disappointment	–	0.186 (*p* < 0.001)
Discouraging teaching approaches	0.248 (*p* < 0.001)	0.170 (*p* < 0.001)

**Table 7:** Direct, indirect, and total effects.

	**Teacher discouraging attitude**	**Discouraging teaching approaches**
Direct effect	0.216*** (E.S = 0.19, medium)	0.170*** (E.S = 0.15, medium)
Indirect effect	0.046*** (E.S = 0.16, medium)	0.046*** (E.S = 0.17, medium)
Total effect	0.262***(E.S = 0.22, medium)	0.216*** (E.S = 0.19, medium)

^***^p < 0.001.

## Discussion

The objective of this study was to examine the direct and indirect impact of teachers' discouraging attitudes and discouraging teaching approaches on student demotivation to learn in English (as a medium of instruction). The findings of this study revealed a significant impact of teacher discouraging attitudes and discouraging teaching approaches on student demotivation. Hence, hypotheses 1 and 2 are supported. These findings indicate that the students are sensitive to teacher-related demotivating factors, as evidenced in this study. Moreover, the results also confirm that when students find their teachers' attitude discouraging, they emotionally negatively react in the form of disappointment with the English language and become demotivated. Therefore, H3a is supported. Similarly, it is yielded that when the teacher used a discouraging teaching approach, it leads to feelings of disappointment with the English language, which in turn decreases the motivation level of students. Hence, H3b is supported.

These findings are according to our expectations and in line with the previous studies (Christophel and Gorham, 1995; Gorham and Millette, 1997; Wang and Littlewood, 2021; Yang and Zhanfang, 2022). The findings also suggest that if universities could alter the negative attitude of their teachers and their teaching approaches, it could be an effective strategy to increase the student's motivation level with English as a medium of instruction. This is because lectures in English can be demanding for Chinese students. When they have a negative experience with their teacher, they ultimately will be less motivated to learn in English. A positive attitude and effective pedagogical approaches can enable the students to learn in English, and embrace the challenges of stress and difficulty associated with English as a medium of instruction. The students will be motivated daily, once they find their teacher with a positive attitude and follows the encouraging teaching approaches (Meng, 2022; Ren and Abhakorn, 2022).

Moreover, students always have some expectations about their teachers. Generally, students consider their teacher as a spiritual father and expect that their teacher will help them in challenging times, and will follow easy ways of teaching. However, when their expectations about their teacher and methods of teaching remained unmet, they become highly disappointed, which is a negative emotional reaction. Our findings have empirically confirmed this phenomenon and found that student disappointment with the English language is an important mediator. These findings are consistent with previous studies (Levering, 2000; Wright, 2013). To overcome this issue, the universities can arrange sessions between students and teachers so that they become aware of each other's issues. Moreover, the management of these universities regularly gets feedback about the teachers' attitudes and teaching methods. If the majority of students are disappointed with the teacher and his or her teaching approaches, the organizations should train or replace that particular teacher. In addition to this, the universities should provide opportunities for Chinese students to practice English so that their proficiency in English can be improved. Doing so can effectively reduce the disappointment of Chinese students, and they will be highly motivated to learn in English (Yu et al., 2021).

Overall, the empirical findings of this study confirmed that students' demotivation with English as the medium of instruction is an important issue in non-English-speaking countries, particularly in China. Considering the role of China in the world economy, it is important for the educational administrators and government authorities to overcome these teacher-related contributing factors. Otherwise, the country will not be able to extract the complete benefits of adopting English as a medium of instruction in educational institutions.

### Limitations and future research direction

The findings of this study are not free from limitations. This study has several limitations that future researchers can address in their research. First, this study is a cross-sectional study, which can lead to common method bias. Hence, future studies can adopt a longitudinal research design and can collect multivalve, multisource data. In addition to this, the study can use an experimental research design to test this model in a more controlled setting. Second, this study has only considered teacher-related determinants of student demotivation with English as a medium of instruction. Future researchers are suggested to analyze the impact of demographic variables, such as educational background and income level. Moreover, researchers can also consider the impact of culture. Third, this study has used emotional mechanisms to identify the indirect effects. Future studies can use alternative mechanisms, including attitudinal reactions in their research model, such as liking English. Fourth, this study has just analyzed the direct and mediating effects and has not considered the moderating factors. Future studies can use some contextual factors, such as the location of the institute, as the moderating variable to get further insights into this important phenomenon. Fifth, future researchers should conduct a comparative study on this issue by collecting data from different non-English-speaking countries. Finally, the study sample was collected from only two cities in China, which can put limitations on the generalizability of the study findings. Future studies can collect data from more cities and universities to overcome this issue.

## Conclusion

Since the motivation of students is an important factor in learning, universities must identify ways to increase students' motivation and decrease demotivation. In this regard, many efforts have been made to explore the determinants of students' demotivation with English as a medium of instruction and the underlying mechanisms. However, the scholars have put little effort into investigating the impact of teachers' discouraging attitudes and discouraging teaching approach on students' demotivation with English as a medium of instruction in China. Similarly, past research has also overlooked disappointment with the English language as an underlying mechanism in this relationship. This study was an attempt to understand the impact of teacher-related factors on student demotivation through the mediating role of disappointment. The present research contributes to the literature on students' demotivation in the Chinese context by identifying teacher-related factors. This is an important contribution that can help the management of universities to specifically work on these factors in order to increase the motivation level of students with English as a medium of instruction. The findings of this study suggest that the universities must work on the discouraging attitude of the teachers and modify their teaching approaches. This will not only reduce students' disappointment but also improve their motivation to learn English. In this way, students' demotivation with English as a medium of instruction among Chinese students can be reduced. This will ultimately help them to learn English and will enable them to deal with global challenges.

## Data availability statement

The original contributions presented in the study are included in the article/supplementary material, further inquiries can be directed to the corresponding author.

## Ethics statement

The studies involving human participants were reviewed and approved by Chengdu Textile Collage, China. The patients/participants provided written informed consent to participate in this study. The study was conducted in accordance with the Declaration of Helsinki.

## Author contributions

JH: conceptualization and data collection. YT: writing the draft. Both authors agreed to the submitted version of the manuscript.

## Conflict of interest

The authors declare that the research was conducted in the absence of any commercial or financial relationships that could be construed as a potential conflict of interest.

## Publisher's note

All claims expressed in this article are solely those of the authors and do not necessarily represent those of their affiliated organizations, or those of the publisher, the editors and the reviewers. Any product that may be evaluated in this article, or claim that may be made by its manufacturer, is not guaranteed or endorsed by the publisher.
